# An Efficient and Precise Remote Sensing Optical Image Matching Technique Using Binary-Based Feature Points

**DOI:** 10.3390/s21186035

**Published:** 2021-09-09

**Authors:** Min-Lung Cheng, Masashi Matsuoka

**Affiliations:** Department of Architecture and Building Engineering, School of Environment and Society, Tokyo Institute of Technology, Yokohama 226-8502, Japan; matsuoka.m.ab@m.titech.ac.jp

**Keywords:** image matching, BRISK, color space, geometric mapping

## Abstract

Matching local feature points is an important but crucial step for various optical image processing applications, such as image registration, image mosaicking, and structure-from-motion (SfM). Three significant issues associated with this subject have been the focus for years, including the robustness of the image features detected, the number of matches obtained, and the efficiency of the data processing. This paper proposes a systematic algorithm that incorporates the synthetic-colored enhanced accelerated binary robust invariant scalar keypoints (SC-EABRISK) method and the affine transformation with bounding box (ATBB) procedure to address these three issues. The SC-EABRISK approach selects the most representative feature points from an image and rearranges their descriptors by adding color information for more precise image matching. The ATBB procedure, meanwhile, is an outreach that implements geometric mapping to retrieve more matches from the feature points ignored during SC-EABRISK processing. The experimental results obtained using benchmark imagery datasets, close-range photos (CRPs), and aerial and satellite images indicate that the developed algorithm can perform up to 20 times faster than the previous EABRISK method, achieve thousands of matches, and improve the matching precision by more than 90%. Consequently, SC-EABRISK with the ATBB algorithm can address image matching efficiently and precisely.

## 1. Introduction

Digital image matching is a technique that searches for homologous feature points, also named matches or correspondences, between two or more images. The generation of spatial products and the wide variety of environmental applications using remote sensing images require such a technique to achieve their goals. For example, image registration [1,2,3,4], object detection and change detection [5], three-dimensional (3D) reconstruction [6,7], mapping tasks [8], and structure-from-motion (SfM) algorithms [9,10] all require a digital image matching stage. Unlike earlier handcrafted operation, advances in technology now allow semi- or fully automatic digital image matching by incorporating computer vision methods, saving both time and labor costs. In addition, several photogrammetric techniques, such as bundle adjustment and image connection, can be carried out more effectively.

Classic automatic image matching techniques can be classified into three categories: area-based matching techniques (ABMs), feature-based matching techniques (FBMs), and hybrid methods [11]. ABMs, also known as template matching, use a window template as a feature point with pixel intensities to compute the feature similarities or resemblances. The typical procedure defines a window template in a master image and moves it across a target image to search for the most similar correspondence. Well-known examples of ABMs include normalize cross-correlation coefficient (NCC), zero-mean NCC [12], least-squares matching (LSM, [13]), and mutual information [14]. Although ABMs can achieve high positional accuracy, e.g., 1/50 pixels according to [15], they may suffer from image occlusions, uniform textures, image distortions, and illumination changes [11]. FBMs extract image feature points of interest, also known as keypoints (e.g., points, lines, and areas), and compute their similarities in different images by using feature descriptors [16,17]. Scale invariant feature transform (SIFT) [18] may be the most representative algorithm in the FBM family. Most FBMs can achieve scale- and rotation-invariant feature descriptors that increase the distinctiveness and robustness among different keypoints; thus, the image matching results can be more precise than those of ABMs [11,19]. However, Sedaghat et al. [11] indicated that FBMs are less accurate than ABMs, and eliminating mismatches/outliers is usually required. Finally, hybrid methods can address the individual limitations of the above two families by combining ABMs and FBMs; otherwise, adding ancillary data such as wavelet information for image matching is also feasible [20]. However, hybrid methods usually require prior knowledge before data processing, so they are not utilized frequently.

More recently, deep learning (DL)-based approaches developed in the field of artificial intelligence (AI) have had great impacts on image matching [21,22,23,24,25,26,27,28,29]. The development of deep features—pre-trained convolutional neural network (CNN) features—is one of the breakthroughs in this study field. However, Refs. [30,31] found that pre-trained CNN features perform similarly or even worse than FBMs. Long et al. [32] tested the capabilities of deep features for semantic alignment and compared the results derived from SIFT and pre-trained CNN features. They concluded that pre-trained CNN features only perform slightly better than the SIFT algorithm. Ufer and Ommer [33] further incorporated a semantic matching algorithm with an object guiding technique into pre-trained CNN features, improving the capacity for deep feature matching. However, pre-trained CNN features mainly concentrate on the salient objects with bounding boxes to constrain the specific regions for matching [23,24,33,34].

Although DL-based methods can achieve very precise results, their requirements, i.e., a large amount of training data and an extensive period for data training, are critical concerns. For example, prior knowledge, such as ground truth and pre-trained CNN features, must be available to implement data training [33,35,36,37]. DL-based methods cannot perform with the lack of such supporting data or when the number of required datasets is inadequate. Revaud et al. [38] proposed the DeepMatching (DM) algorithm and compared its performance with the SIFT method. In terms of memory usage, they reported that the DM algorithm consumed approximately 100 times more than the SIFT method when utilizing the MPI-Sintel dataset and the KITTI dataset (9.9 GB versus 0.2 GB). As for the matching time, the DM algorithm spent 8.1 min, while the SIFT method required only 1.4 s when using the Mikolajczky dataset. After investigating the performance among classical approaches and the LF-Net with SuperPoint models [23,24], Bojanić et al. [39] concluded that their DL-based model indeed does not significantly improve the image matching results compared to classic approaches. Bhowmik et al. [40] also indicated that the high precision is caused by matching low-level features rather than improving the image matching performance itself. In addition, the applicability of the prediction map generated by a specific training dataset is limited to the related imagery data only, and their high computational complexity also inhibits the practical application of DL-based methods [41]. Indeed, classic image matching approaches are still favorable in terms of their universality and practicality [42,43], particularly FBMs.

Image matching exploiting FBMs, also known as local feature matching, undergoes three steps: feature detection, feature description, and feature matching [44]. The feature character using FBMs can be further divided into two types: vector-based features and binary-based features. Matching vector-based features utilizes Euclidean distance and a ratio test to determine the resemblance between two keypoints [18]. In addition to SIFT, several variations, such as principal component analysis SIFT (PCA-SIFT) [45], gradient location-orientation histogram (GLOH) [46], speed up robust features (SURF) [47], affine SIFT (ASIFT) [48], uniform robust SIFT (UR-SIFT) [11], and adaptive-binning SIFT (AB-SIFT) [44], are also included in the family of vector-based features. These methods either enhance the feature distinctiveness or reduce the dimensions of the feature descriptors to improve the performance of SIFT-like image matching. On the other hand, binary-based features employ the Hamming distance [49], an XOR operation, to evaluate the similarity between two keypoints. Approaches in this category include binary robust independent elementary features (BRIEF) [50], orientation FAST and rotated BRIEF (ORB) [51], and binary robust invariant scalable keypoints (BRISK) [52]. As the ORB technique lacks the trait of scale invariance [53], the BRISK technique can be considered the most powerful method in the family of binary-based features because it is scale- and rotation-invariant. Based on this technique, Liu et al. [54] fused the depth information into the BRISK feature descriptor to enhance the scale invariance using a specific camera for capturing depth information with optical images simultaneously, leading to the BRISK-D algorithm. One of the most significant advantages of this approach is that it can perform image matching properly under illumination changes. However, they observed that the precision of the image matching results decreases when the image has a large-scale change, and the algorithm may be unstable when using blur images. Additional modifications, such as the accelerated BRISK (ABRISK) [4] and enhanced ABRISK (EABRISK) [55] algorithms, further improve the performance of image matching using BRISK in terms of the data processing time and the number of matches.

Tsai and Lin [4] compared the capacities among the SIFT, SURF, and ABRISK algorithms, discovering that the ABRISK method can perform 312 times and 202 times faster than the SIFT and SURF methods, respectively, when using the image size of 4000 × 4000. They concluded that vector-based features provide more robust results, but they consume more time for data processing; on the contrary, binary-based features take less time for image matching, and the outcomes are acceptable. Their results also indicated that the number of matches becomes sparser, implying the inability to obtain redundant correspondences for more rigorous geometric computation when performing spatial tasks such as image registration and SfM. Similarly, Kamel et al. [53] compared the results by utilizing hybrid features with the airport dataset and found that ORB-BRISK requires 0.238 s for 37 matches and SURF-SFIT consumes 3.518 s for 161 matches, respectively. Shao et al. [56] also utilized a hybrid method by integrating SIFT and ASIFT to improve the accuracy of the image matching results for land monitoring. Cheng and Matsuoka [55] further improved the ABRISK by incorporating the human retina mechanism and showed that the EABRISK reduces the data processing time by approximately 10%. In addition, EABRISK can achieve approximately 1.732 times more matches than ABRISK when applying drone image pairs.They also explored the performances of the AB-SIFT and EABRISK methods, showing that these two algorithms almost have a comparable data processing time and number of matches obtained. For several practical cases, EABRISK can provide better image matching results than AB-SIFT.

Currently, most image matching algorithms convert an optical image into a grayscale image and utilize pixel intensities to match different images; color information, namely the red, green, and blue frequency bands, is not involved. A few methods, such as the colored SIFT (CSIFT) [57] and colored BRISK (CBRISK) [58] techniques, use a spectrum model to normalize color spaces to generate color-invariant images and thus avoid the influences of different illumination conditions caused by radiometric changes. However, both techniques may alter the true electromagnetic information stored in the original imagery data, thus leading to some mismatches with unknown causes. Alitappeh et al. [59] indicated that such color-invariant techniques are only suitable for specific cases. The color-based retina keypoints (CREAK) method [60], based on the fast retina keypoint (FREAK) technique [61], performs feature detection and assesses descriptor changes in the red, green, and blue (R-G-B) color spaces. These three color spaces have distinct impacts on feature detection and descriptor formation and therefore should be treated separately in image matching.

For stereomatching that has two images only, the DL-based approaches may not be suitable to address the issue because the ground truth is not known and the number of training datasets is not sufficient. In most past studies, FBMs are crucial in solving this issue. To further improve the performance of stereomatching, this paper develops an integrated approach with two steps to achieve substantial precise matches and balance the image matching efficiency. The first step exploits the EABRISK method as the fundamental by considering its data processing efficiency and the matching result obtained. Different from the EABRISK method using the grayscale image, this research further adds color information into the feature descriptors to increase the distinctiveness and robustness for image matching. Instead of applying the spectrum model to normalize color spaces, this study utilizes R-G-B images and simulates the human retina mechanism to achieve the purpose. The second phase aims to increase the number of matches for stereomatching by geometric mapping since FBMs can usually yield sparse results. By this means, the feature point detected has an opportunity to find its correspondence to increase the number of matches. The rest of this paper is organized as follows. Section 2 describes the proposed methodology in this research. Section 3 demonstrates and analyzes the experimental results by using imagery datasets with different conditions. Section 4 discusses the abilities and the limitations of the proposed method. Section 5 draws the conclusions and proposes future works so that further improvements can be made.

## 2. Materials and Methods

**Figure 1:**
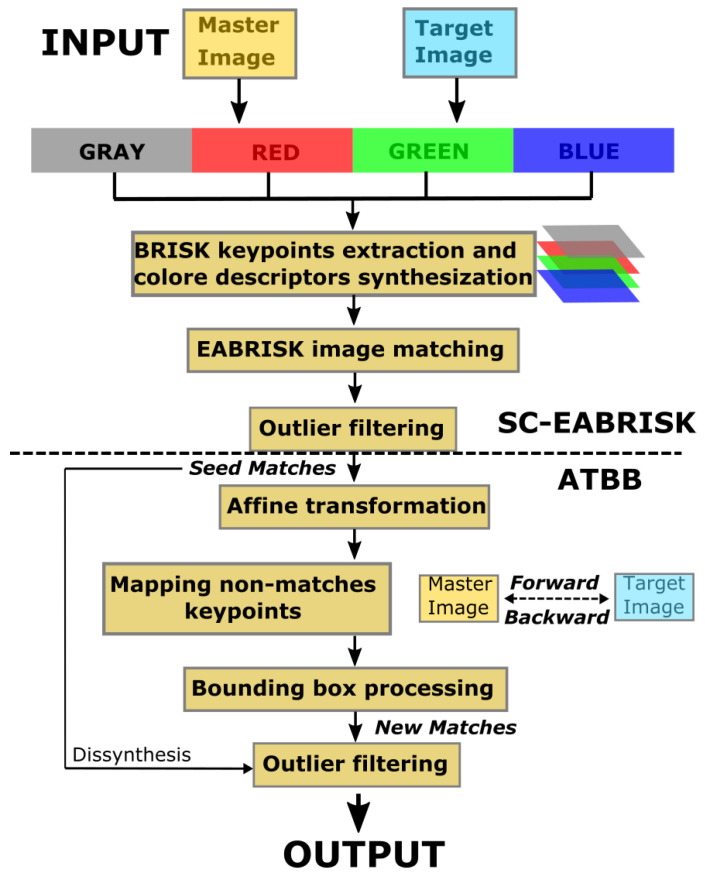
The proposed two-step workflow for image matching.

Building on BRISK-based methods, this paper proposes an integrated remote sensing image matching algorithm to achieve as many feature correspondences as possible while balancing the data processing time via a two-step process. Figure 1 presents the complete workflow of the proposed methods. The purpose of the first step is to select the most representative keypoints in an image and add color information into the feature descriptors to increase their robustness for more precise image matching. The second step is an extension whose goal is to retrieve more feasible correspondences from the keypoints skipped in the first step. Based on the proposed schema, this research intends to balance the time consumed for image matching and the number of matches that can be obtained. Google Colab was used in this research to process the imagery data without using the graphical processing unit (default setting).

### 2.1. Enhanced Accelerated BRISK Algorithm

The EABRISK algorithm developed by Cheng and Matsuoka [55] was intended to improve the efficiency of the BRISK technique in image matching and retrieve more feature correspondences from keypoints of high similarity. This method includes two parts: the inverse sorting ring (ISR) and the interactive two-side matching (ITSM) approaches.

**Figure 2:**
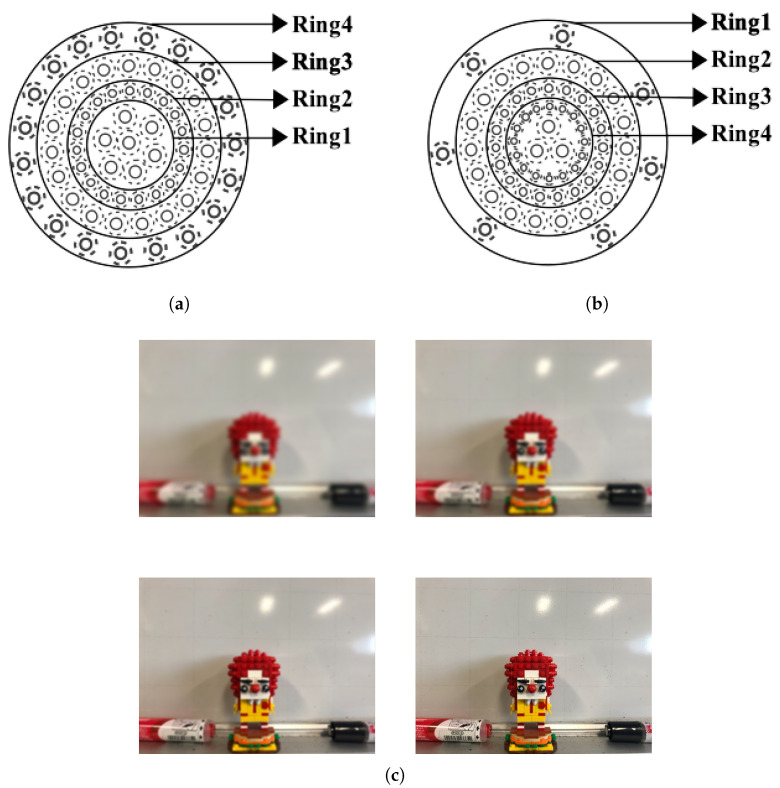
Separating 64-byte BRISK feature descriptors into four rings using grayscale image: (**a**) the sorting ring pattern, (**b**) the inverse sorting ring pattern, and (**c**) scene changes from blurred to clear.

The ISR approach simulates the function of the human retina [62] and the mechanism of visual accommodation [63] to increase the efficiency of image matching. Different from the sorting ring (SR) pattern shown in Figure 2a [4], the ISR pattern exhibited in Figure 2b [55] redistributes the 64-byte feature descriptors into groups of 7, 19, 19, and 19 bytes from the outermost to the innermost ring, based on the distribution of ganglion cells across the human retina [64,65]. For image matching, the mechanism of visual accommodation shown in Figure 2c is applied to the ISR pattern, where feature similarities are evaluated progressively from the outermost ring to the innermost ring by the Hamming distance. The goal of this ring-by-ring process is to find and eliminate unlikely matches in the early stages so that the feature similarities for inner rings do not need to be computed. According to ABRISK [4], the thresholds determining the feature similarities in terms of the Hamming distance are set to 18, 35, 40, and 45 from the outermost ring to the innermost ring, and a threshold of 80 is used as the last step to evaluate the entire similarity of two feature points. As a result, the ISR approach can perform image matching more efficiently by rearranging the 64-byte feature descriptors based on the operation of the human eye.

As feature points of very high similarity cause ambiguities in determining the most likely match, the ITSM strategy attempts to address these ambiguities to achieve more matches by selecting the most likely match based on the minimal Hamming distance within a group of possible matches of very high similarity. For the case of stereomatching, the most likely match is further determined by the use of forward and backward processes and cross-checking for its consistency, thus reducing the ambiguities. Consequently, the ITSM strategy can retrieve the missing matches from those of very high similarity and increase the number of feature correspondences. Through the interoperability of ISR and ITSM, the EABRISK algorithm can address image matching effectively [55].

### 2.2. Synthetic-Colored Feature Descriptors

Converting an optical image into a single grayscale channel is the most widely adopted approach for performing image matching, but the R-G-B color information is neutralized. According to [60], however, a feature point in a grayscale image may behave differently in the R-G-B channel spaces in terms of the properties extracted in the feature detection stage that computes scale and orientation invariances. This finding also implies that the feature descriptors of the feature points acquired from different color channels can be different. Color information, hence, can be considered useful for supporting the grayscale image for increasing the distinctiveness and robustness of the feature point.

**Figure 3:**
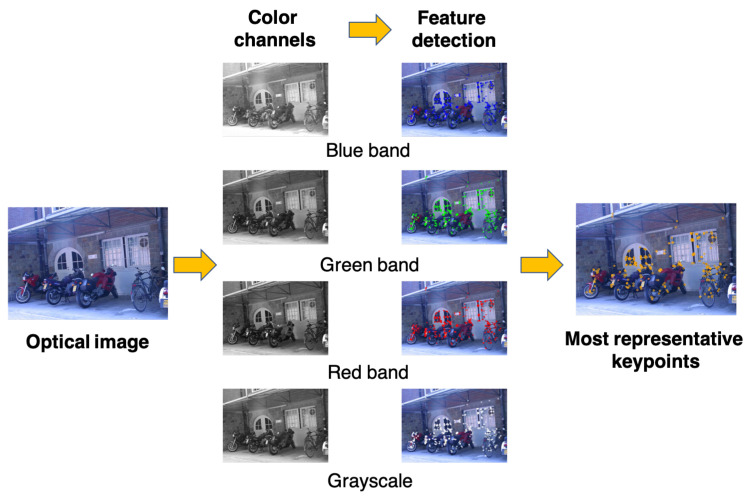
Detecting BRISKs in grayscale and R-G-B channels and finding common keypoints in the four images (image accessed from https://www.robots.ox.ac.uk/~vgg/data/affine/, accessed on 23 January 2021).

**Figure 4:**
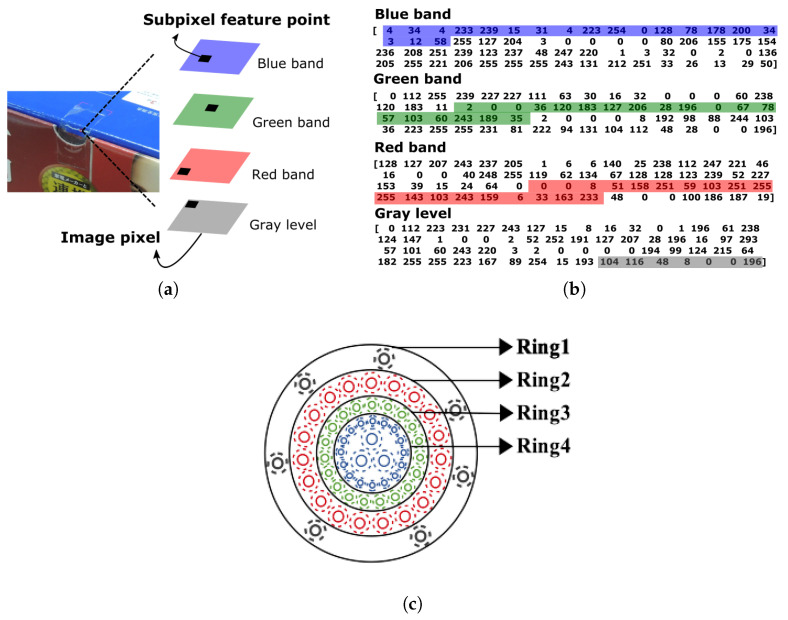
Synthesizing colored feature descriptors for the most representative keypoints. (**a**) Detecting the most representative keypoints, (**b**) extracting color descriptors, and (**c**) inserting synthetic-colored feature descriptors into the ISR pattern with R-G-B color information.

As presented in Figure 3, it is apparent that the BRISKs detected in the separated color channels are different, and some feature points that emerge in one channel may not be available in the other channels. Instead of using all detected feature points for image matching, this paper aims to select the most representative keypoints that appear in all four channels based on their highest repeatability. However, due to the effects of feature detection and computation at the subpixel level, it may not be possible to obtain completely identical feature points in the four channels. To solve this issue, this study utilizes a nearest-neighbor strategy based on position to determine the most representative keypoints. Figure 4a illustrates the procedure of determining a desirable feature point in all four channels, where the nearest-neighbor strategy handles the inconsistencies in terms of subpixel-level positions. As shown in the figure, the method proposed in this paper associates the four feature points that emerge in all four channels and combines them as a single feature point; consequently, a group of the most representative keypoints can be generated.

In terms of the nearest-neighbor strategy, this study gathers four feature points from the four channels based on their image coordinates in which their row and column count are identical. This strategy simplifies the image coordinates to integers to acquire the maximum number of SC keypoints; otherwise, subpixel-level coordinates with complex decimals may reduce the number of SC keypoints. For example, four sets of image coordinates—(213.8, 500.4), (213.6, 500.2), (213.6, 500.1), (213.9, 500.3)—available in the four channels can be gathered as an SC keypoint when simplifying their image coordinates to integers.

After determining the most representative keypoints, the proposed method further reforms the feature descriptors by adding the color information. Since each individual most representative keypoint is composed of four channels, four sets of 64-byte feature descriptors are available. According to Hendrickson [66], cells in the human retina are arrayed in discrete layers that can be simplified into four orders—rods, red cones, green cones, and blue cones—which can be considered to correspond to the four image color channels: grayscale, red, green, and blue. Because the ISR pattern involves four concentric circles, which also contains four rings in which the feature descriptors are arranged, the proposed method assumes that each ring is responsible for the information of an individual color channel.

Since the EABRISK algorithm evaluates feature similarities from the outermost ring to the innermost ring as a way to imitate human visual accommodation, the outermost ring should contain a mixture of information that has the least visual impact. As shown in Figure 4b, the last seven feature descriptors derived from the grayscale image are placed into the outermost ring, which is similar to the rods within the human retina. Following the retina cell order described above, the ring just within the outermost ring is responsible for red cones, and thus, the 19 feature descriptors belonging to this ring are derived from the red channel. This process is continued until the four rings are filled with the required descriptors corresponding to their color channels. However, it should be noted that the positions of the 7, 19, 19, and 19 feature descriptors to be extracted from the four color channels should be consistent with their corresponding positions in the new ring. Consequently, the 64-byte feature descriptors of each individual most representative keypoint are synthesized with their color information, and thus synthetic-colored keypoints (SC keypoints), as shown in Figure 4c, are produced for EABRISK image matching (SC-EABRISK). Figure 2 and Figure 4c show comparisons of the distributions of feature descriptors and their compositions among the ABRISK, EABRISK, and SC-EABRISK methods.

### 2.3. Geometric Mapping for Additional Matches

One of the disadvantages of matching local feature points is that the number of matches may not be as large as expected. This can occur due to either: (1) the recognition and extraction of only certain feature points (e.g., corners) by the future detection algorithm or (2) the lack of identical keypoints between the two images. Although many image processing algorithms require only a portion of matches to address the demands of the system, more matches are often needed to improve the precision or reliability of the outcomes. For example, in affine image registration, at least three matches are required to solve for six parameters mathematically. With the inclusion of extra matches, the least squares method helps to improve the precision of the six parameters. The consideration of the spatial distribution of the matches within the images is also important because matches that form weak geometric networks may produce unstable solutions. For instance, if only three matches are used for affine image registration, they must not be collinear; otherwise, the six affine parameters cannot be determined. With more matches available, there is a higher probability of achieving a better geometric network. Such a concern is also pertinent to the eight-point [67] and five-point [68] algorithms used for the SfM problem and relative orientation parameter (EOP) estimation in photogrammetry. For practical uses and applications, therefore, obtaining additional matches is an important requirement for ensuring more reliable results.

**Figure 5:**
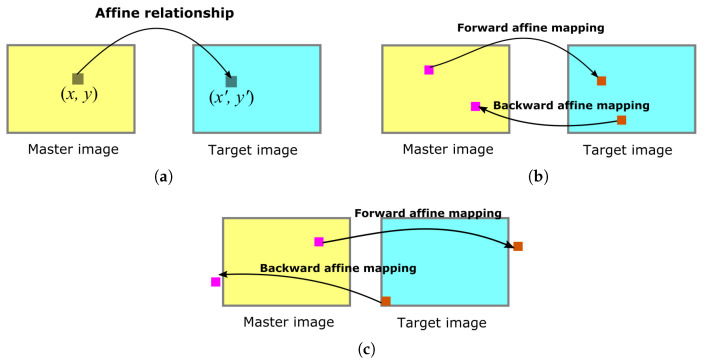
Affine transformation and bounding box processing for retrieving a high number of feature correspondences: (**a**) solving the affine parameters by seed matching, (**b**) geometrically mapping the keypoints, and (**c**) filtering matches outside the images.

As the SC-EABRISK algorithm selects the most representative keypoints to perform image matching, it is apparent that the number of matches obtained must be reduced. To compensate for this detriment, the proposed method further exploits geometric mapping to retrieve feasible matches from the keypoints that are not used during the SC-EABRISK image matching stage. An important reason for addressing geometric mapping is to avoid spending extra data processing time to maintain the efficiency of the entire process. For a given stereo pair involving a master image and a target image after SC-EABRISK processing, the results—the seed matches—are utilized as control points (CPs) to determine the geometric relationship between the two images. The proposed method in this study uses affine transformation according to Equation (1) as the geometric mapping function, conditioned upon guaranteeing that at least three matches can be derived from the SC-EABRISK processing to solve the six affine parameters (*a*, *b*, *c*, *d*, *e*, *f*), as described in Figure 5a. When there are more than three CPs, the least-squares method is used to compute more precise affine parameters.

(1) $$\begin{array}{c} x^{\prime }=ax+by+c\\ y^{\prime }=dx+ey+f \end{array}$$

When mapping unused keypoints, the proposed method incorporates all keypoints emerging from the four channels in both the master and target images to obtain the greatest number of correspondences possible. As shown in Figure 5b, the geometric mapping function obtained by using the six affine parameters therefore maps from the master image to the target image, and vice versa. This process provides each unused keypoint the opportunity to find its correspondence; however, it is important to note that this implementation may cause some feature points to be mapped outside the image. To address this issue, the proposed method utilizes a bounding box based on the dimensions of the image (rows and columns) as the boundaries to filter any invalid feature points mapped, as demonstrated in Figure 5c. As a result, every keypoint detected can find their most likely correspondences by the affine transformation and bounding box (ATBB) procedure, thus extensively increasing the number of matches when carrying out stereomatching.

### 2.4. Outlier Removal and Evaluation Indicators

Detecting and removing mismatches (outliers) is typically the last step for most FBMs, after which correct matches (inliers) can be obtained and preserved. Instead of manual outlier removal, automatic algorithms based on sample consensus are usually applied to determine the greatest number of correct matches (NCMs).

Random sample consensus (RANSAC), proposed by Fischler and Bolle [69], may be the most prevalent method due to its simple but useful assumptions. RANSAC randomly selects four matches from all available data to estimate the spatial relationship between two images, e.g., homography or affine transformation. The spatial relationship is thereafter considered a fitting model, and the remaining matches are assessed to test the capacity of the fitting model by using linear regression and a prespecified threshold. Through iterative testing of several fitting models, RANSAC finally provides the best result that achieves the greatest NCMs under the given threshold. M-estimator sample consensus (MSAC) is a method similar to RANSAC but is dependent on the threshold itself rather than the greatest NCMs that can be obtained [70,71].

Locally optimized RANSAC (LO-RANSAC) is an extension that further optimizes the current best fitting model iteratively to recognize additionally probable outliers from the RANSAC result [72], allowing an optimal fitting model to thus be determined for all matches. Although the NCMs obtained may be reduced, LO-RANSAC increases the quality of the results while maintaining comparable efficiency to RANSAC in terms of data processing. Similar to RANSAC, this algorithm also needs a given threshold to discriminate inliers and outliers.

In addition to the above methods, advanced approaches known as universal sample consensus (USAC) approaches are demonstrating impressive effectiveness in solving this task [73]. Graph-Cut RANSAC (GC-RANSAC), devised by Barath and Matas [74], is a local optimization method utilizing energy minimization and spatial coherence to divide inliers and outliers; specifically, it globally refines the so-far-the-best fitting model so that the final outcomes are stable and precise. Similar to RANSAC and LO-RANSAC, GC-RANSAC also requires a threshold to separate inliers and outliers. In contrast, marginalizing sample consensus (MAGSAC) is an entirely threshold-free algorithm based on σ-consensus [75]; it progressively marginalizes outliers and attempts to determine the greatest NCMs. To prevent the given threshold from influencing the final results, the proposed method in this study utilizes the MAGSAC algorithm to remove outliers; in Figure 2, outlier removal is performed twice to determine the CPs for the ATBB method and to further improve the precision of the final output.

The performance of the proposed SC-EABRISK with the ATBB method is assessed via four indicators: the NCMs obtained, matching precision (MP), recall, and effectiveness. In many image matching studies, the NCM, derived from outlier removal, is a straightforward indicator for evaluating the algorithm. MP, defined by Equation (2), is the ratio of the NCMs over the number of total matches [44,46,76,77]. The recall, explained by Equation (3), describes the ability of the image matching algorithm in identifying the NCMs out of all possible matches (APMs) in the original imagery data, where the APMs are determined by the ground truth and homography [46,78,79]. Due to the lack of such prior knowledge, this study instead selects the APMs by the smallest number of keypoints detected in either one of the images. Because FBMs only allow one-to-one feature correspondence, it is apparent that the smallest number of keypoints detected dominates the APMs. Therefore, it should be noted that the definitions of MP and recall used to assess the performance of the image matching algorithms are different from those used in the confusion matrix adopted by AI studies. The last indicator of effectiveness, shown in Equation (4), evaluates the efficiency of the algorithm, and is calculated by the NCMs over the time consumed (TC) [55].

(2) $$MP=\frac{NCMs}{NCMs+outliers}\times 100\%$$

(3) $$Recall=\frac{NCMs}{APMs}\times 100\%$$

(4) $$Effectiveness=\frac{NCMs}{TC}$$

## 3. Experimental Results and Analysis

The experimental results present and analyze the performance and generalizability of the proposed algorithm by using three kinds of imagery datasets. The first dataset contains eight benchmark images (four pairs) accessed from the INRIA Rhone-Alpes research center to test the preliminary ability of the proposed method. The second dataset involves close-range photos (CRPs) that frequently address remote sensing issues such as 3D modeling. The third dataset presents aerial and satellite image pairs that aim for remote sensing tasks such as large-scale environmental monitoring. Although the image dimensions are a factor that affects the result of the image matching, this paper instead focuses on the number of features extracted and synthesized for image matching and analysis. In addition, this research also compares the image matching results with two relevant approaches building on ORB—learned arrangements of three patch codes (LATCH) [80] and boosted efficient binary local image descriptor (BEBLID) [81]—to further investigate the performance of the proposed method. These two approaches attempt to improve the binary feature descriptors to make them more distinctive. The LATCH method compares the intensity of three-pixel patches surrounding a given ORB keypoint to reproduce the binary descriptors, and the BEBLID approach harnesses Adaboost to modify the binary descriptors of ORB keypoints.

### 3.1. Experiments and Analyses on Benchmark Imagery Datasets

**Figure 6:**
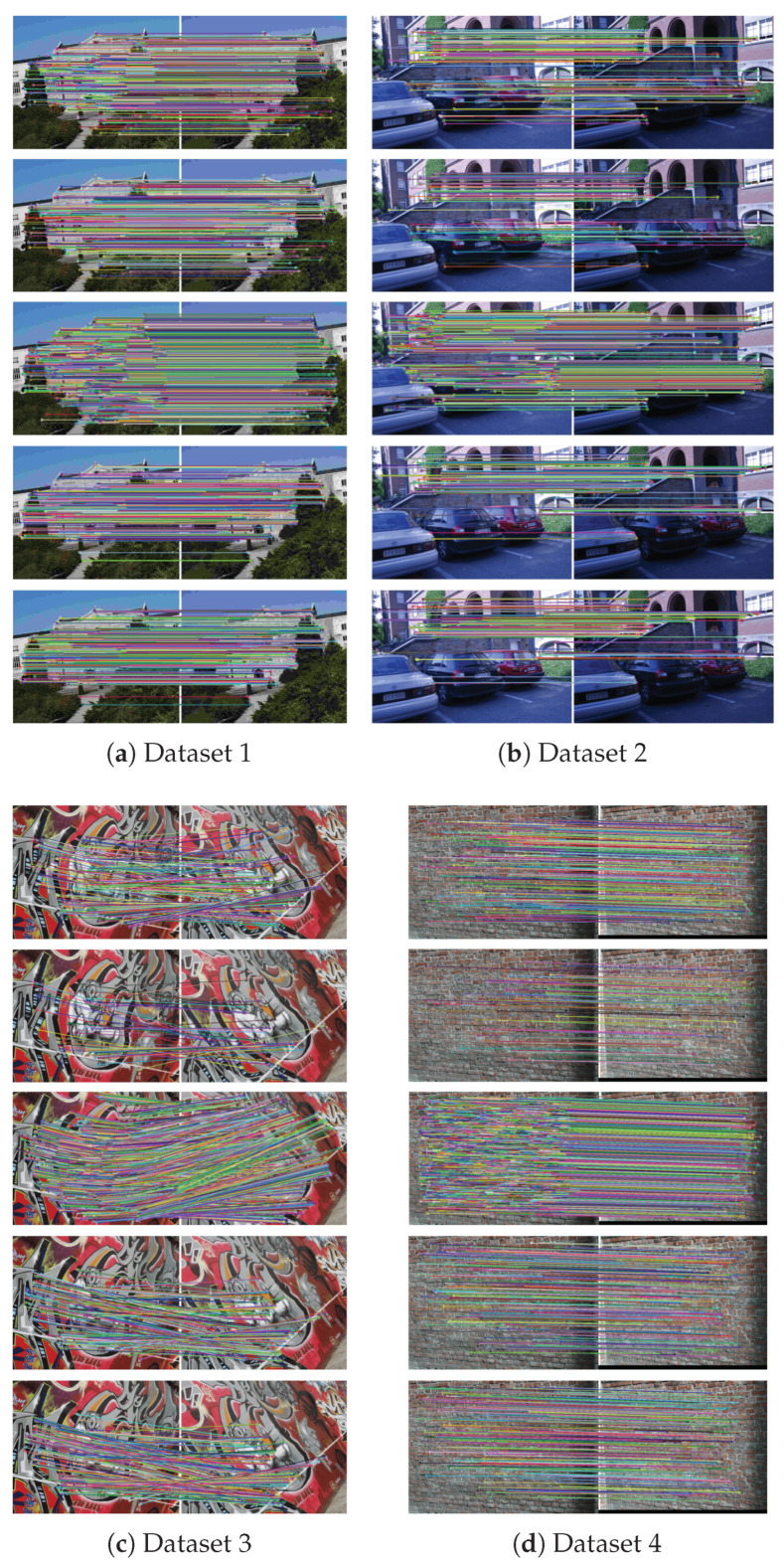
Matching images by using four benchmark imagery datasets demonstrating different variations with five algorithms (from top to bottom: EABRISK, SC-EABRISK, SC-EABRISK with ATBB, BEBLID, LATCH).

Four image pairs were randomly selected from the six benchmark datasets involving 48 images. Figure 6 presents the results of the image matching from the five methods: EABRISK, SC-EABRISK, SC-EABRISK with ATBB, BEBLID, and LATCH. Each image pair shows different characteristics as follows: dataset 1 has different image resolutions, dataset 2 has varied illumination conditions (radiometric changes), dataset 3 contains image distortions, and dataset 4 demonstrates uniform textures. All the outcomes are presented following outlier removal by the MAGSAC approach. Compared with the EABRISK method, the SC-EABRISK approach reduces the NCMs because of the number of SC keypoints utilized, implying that the the post-processing of ATBB is needed. By using the NCMs derived from SC-EABRISK as CPs, the ATBB procedure helps to retrieve more feasible feature correspondences. In addition, the quantity and spatial distribution of the CPs address the six affine parameters, proving the assumption that the more matches there are, the higher their probability of being evenly distributed across the image. These results also indicate that the SC-EABRISK with ATBB method can acquire more feature correspondences compared to the BEBLID and LATCH methods, showing that the features extracted from the four channels can contribute additional feature matches.

Table 1 shows the number of features detected and extracted from the grayscale and R-G-B images and the number of feature pairs (FPs) used in the the data processing step. In this experiment, both the BEBLID and LATCH approaches utilize grayscale images to perform feature matching. In addition, Table 2 presents the TC by using the five algorithms. Different from the SC-EABRISK approach, the EABRISK method utilizes mainly pairs of grayscale images to carry out image matching. As the BEBLID and LATCH methods mainly focus on improving the distinctiveness of the feature descriptors instead of the matching algorithm, their TCs are not included for comparison. Based on these results, it is evident that the number of SC keypoints is significantly reduced for all image pairs, resulting in a substantial suppression of the execution time. Based on these results, the proposed method can perform image matching more efficiently because the TC is significantly reduced. In this experiment, the TC for the SC-EABRISK approach is less than that of the EABRISK method by approximately eightfold at maximum (i.e., dataset 3).

**Figure 7:**
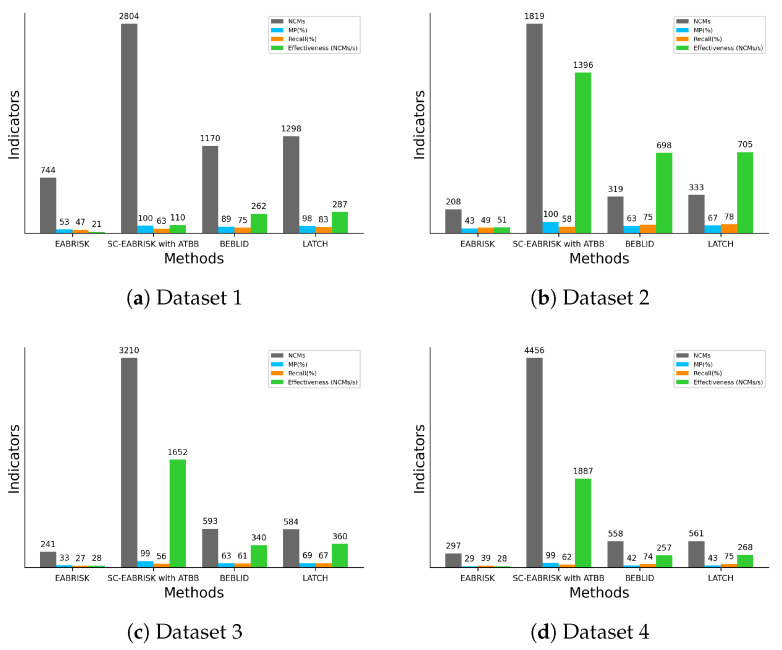
Numerical analyses of the image matching results by using the benchmark imagery datasets.

To assess the performance of the proposed method, this paper compares the results derived from the EABRIAK, SC-EABRIAK with ATBB, BEBLID, and LATCH algorithms separately and investigates their differences in terms of NCMs, MP, recall, and effectiveness. Figure 7 presents the numerical analysis of the image matching results. Figure 7a shows that both the BEBLID and LATCH methods present better recall and efficiency values than the SC-EABRISK with the ATBB method, but the proposed schema can acquire more feature correspondences in this case. For the remaining results, this study investigates that the proposed SC-EABRISK with ATBB method shows better performance as the imagery scenes become more complex. In addition, the recall values obtained by using the SC-EABRISK with ATBB method may not be very high (e.g., 100%) because some of the matches are filtered either by the bounding box or via outlier removal. Therefore, the recall values in these experiments range from 50% to 60%, meaning that half of the keypoints within an image pair can be matched to their correspondences successfully. The high recall values presented by the BEBLID and LATCH approaches in all image pairs show their ability to improve the feature descriptors. Based on these experiments, this study also observes that both the BEBLID and LATCH approaches have approximately comparable performance for image matching.

### 3.2. Experiments and Analyses on CRPs

Since CRPs are the most widely used imagery dataset for performing SfM-based 3D reconstruction, applying the proposed algorithm to CRPs is also important. Although there is no clear definition of CRPs, this paper classifies them as ground-based and drone images because the distances between the scene object and the camera in these images are much shorter than in aerial and satellite imagery. The first case presents a pair of drone images capturing buildings devastated by the Kumamoto earthquake in Japan in 2016. A surveying team from Chiba University collected disaster images a few days after the earthquake to reconstruct the site on a computer for spatial and environmental analyses. The second example includes two ground-based CRPs accessed from images courtesy of Carl Olsson for a standard SfM problem. In both cases, local feature matching plays an essential role in establishing the spatial relationships among images, namely the estimation of ROPs.

**Figure 8:**
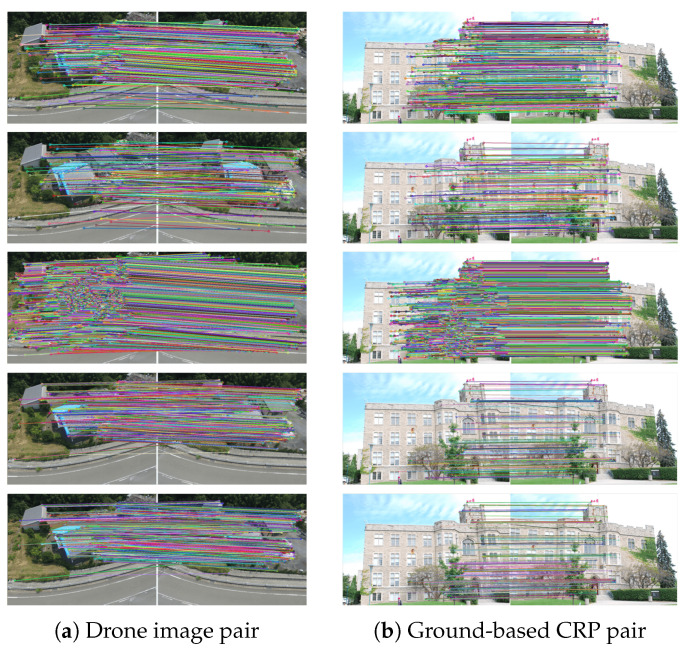
Image matching by using CRPs (from top to bottom: EABRISK, SC-EABRISK, SC-EABRISK with ATBB, BEBLID, LATCH).

Table 3 documents the number of keypoints extracted from the grayscale and R-G-B images and the number of SC keypoints, and Table 4 shows the TC by using the three algorithms. Similar to the results obtained with the benchmark imagery datasets, these tables show that the SC-EABRISK algorithm can reduce the data processing time by up to approximately tenfold, while the ATBB uses two seconds to geometrically map unused keypoints and find their correspondences. Therefore, image matching with the proposed method is more efficient than the previous EABRISK algorithm in terms of effectiveness. In addition, Figure 8 shows the image matching results after outlier removal with the five approaches. Similar to the previous experiments, the number of NCMs obtained with the SC-EABRISK method decreases, after which the ATBB implementation helps to increase it. Moreover, both the BEBLID and LATCH methods present comparable image matching results in terms of ORB detected, implying their similar performances. However, the proposed SC-EABRISK with ATBB algorithm can obtain more NCMs evenly distributed in the images.

**Figure 9:**
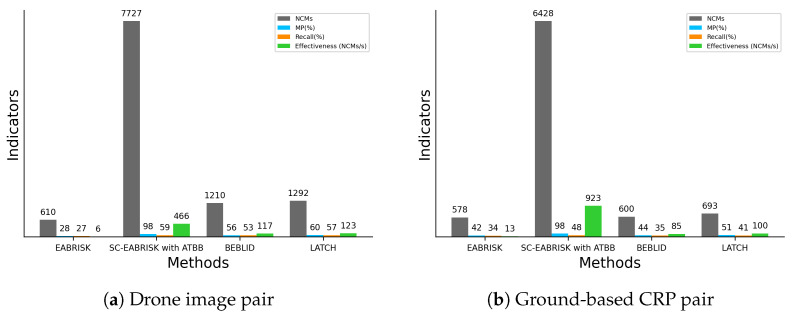
Numerical analyses of the image matching results obtained with CRPs.

Figure 9 presents the numerical analyses of the four indicators to evaluate the performance of the proposed algorithm. Based on the results, the SC-EABRISK with ATBB method shows better performance than the EABRISK, BEBLID, and LATCH approaches in terms of the NCMs, MPs, and efficiency. Similarly, the BEBLID and LATCH methods show similar capabilities for image matching when using the CRP dataset. In these two imagery datasets, however, the recall values of all four methods become lower than the benchmark datasets. Because the CRPs are captured from different positions with different viewing angles, the correspondences of some keypoints are not available. In addition, the impact of uniform textures may lead to mismatches when the viewing position and angle between the two CRPs are different. However, the proposed SC-EABRISK with the ATBB method can still achieve thousands of matches in both CRP pairs and improve the efficiency values.

### 3.3. Experiments and Analyses on Aerial and Satellite Images

In addition to the above experiments and analyses, this paper also applied the proposed method to a pair of orthoaerial images with 80% overlap to examine its performance in the case of image registration, because such a task requires feature correspondences for CPs to estimate the spatial relationship between the two images. A pair of IKONOS satellite images with different illumination conditions were also utilized to examine the performance of the developed method.

Table 5 shows the number of keypoints detected in the grayscale and R-G-B images and the number of SC keypoints produced with both imagery datasets. It can be observed that the number of SC keypoints decreases substantially for the orthoaerial images, while for the satellite images, approximately 60% of the keypoints are preserved with respect to the original data. Table 6 records the data processing time of the three algorithms for two datasets, showing that, for the orthoaerial images, the SC-EABRISK algorithm reduces the data processing time by approximately 37 times with respect to the EABRISK method. However, it is also evident that the NCMs also decreased drastically due to the reduction in the number of SC keypoints. In contrast, the satellite images do not result in such dramatic outcomes because the number of SC keypoints is moderately maintained, so the SC-EABRISK algorithm reduces the processing time by approximately 2.7 times. The implementation of the ATBB method for both cases requires approximately 1 to 2 s to process the unused keypoints to find their correspondences. Considering the entire data processing time, the SC-EABRISK integrated with the ATBB algorithm is up to 20 times faster than the previous EABRISK approach.

**Figure 10:**
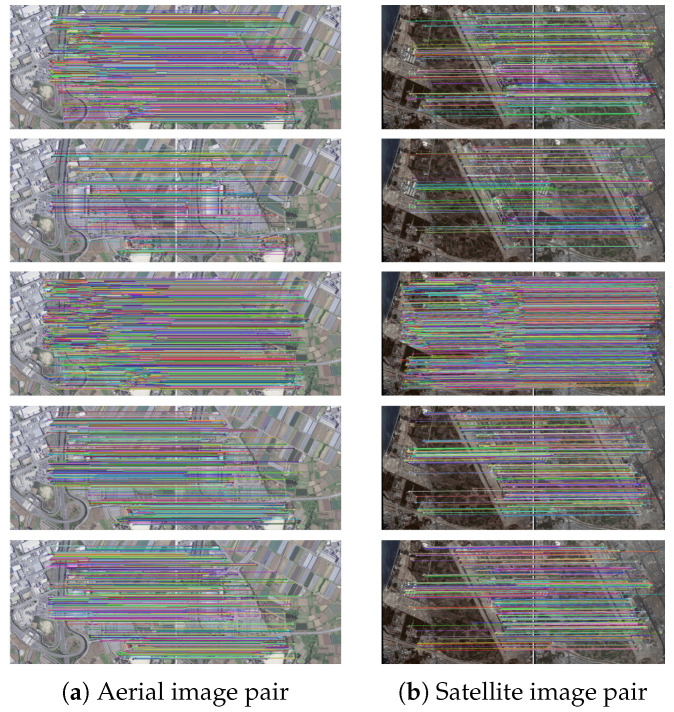
Image matching with using aerial and satellite image pairs (from top to bottom: EABRISK, SC-EABRISK, SC-EABRISK with ATBB, BEBLID, LATCH).

Figure 10 visualizes the image matching results for the imagery datasets used. Similar to the previous results, the number of NCMs is apparently reduced with SC-EABRISK, but the implementation of the ATBB then increases it. According to the results in Figure 6b and Figure 10b, the illumination change between two images may influence the NCMs derived from the SC-EABRISK; therefore, the ATBB process can effectively compensate for this disadvantage. In addition, the results derived from the BEBLID and LATCH methods are similar for both image pairs. This study also observes that the distribution of the matches of the satellite image is more uneven than that of the aerial image pair. Because the grassland in the middle of the satellite image pair presents minor texture variations, feature points may be drastically reduced in this area.

**Figure 11:**
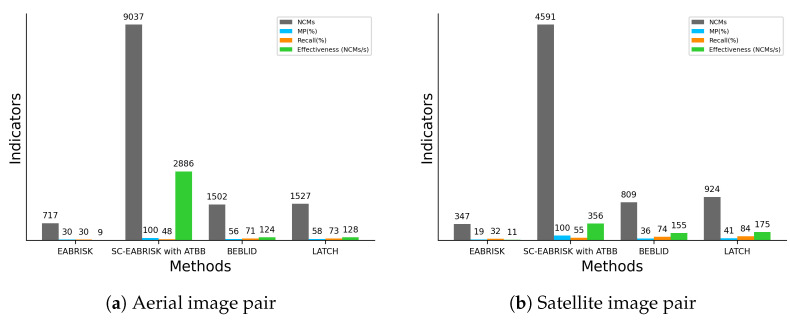
Numerical analyses of the image matching results by using aerial and satellite image pairs.

Figure 11 describes the quantitative analyses of the experimental results. Similar to the previous results, both the BEBLID and LATCH methods present comparable performances in terms of all indicators. For both image pairs, the proposed SC-EABRISK with ATBB algorithm shows approximately fourfold to fivefold more NCMs compared to the BEBLID and LATCH approaches because of the involvement of all keypoints detected in the color channels. When performing orthoimage registration, the proposed SC-EABRISK with ATBB approach can not only provide redundant CPs to conduct the least-squares calculation but also stabilize and improve the precision of the transformation parameters. In terms of effectiveness, the proposed SC-EABRISK with ATBB algorithm presents better performance than both the BEBLID and LATCH methods. This observation is consistent with the results derived from the benchmark datasets and CRPs, proving the high efficiency of the proposed method.

## 4. Discussion

Automatic remote sensing optical image matching is fundamental and crucial for many spatial applications. In addition to the reliability and robustness of the local features themselves, the efficiency of the data processing step has gained more attention in recent years [4]. This paper aimed to develop a systematic workflow that is able to adopt a portion of the most robust keypoints and a subsequent geometric mapping to generate the greatest number of feature correspondences. Different from previously advanced studies that used only grayscale images for feature detection and descriptor formation [4,44], this study aimed to also synthesize color information extracted from R-G-B images into the feature descriptors acquired from the grayscale image to improve their robustness by utilizing BRISKs.

**Figure 12:**
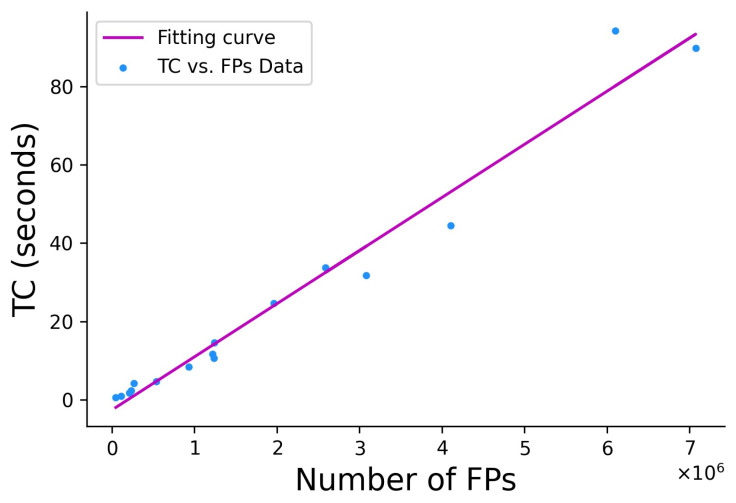
FPs versus TCs curve fitting.

However, the displacements of possibly identical keypoints caused by subpixel-level BRISK detection and extraction in the four images prevent direct color synthesis. To achieve this objective, the proposed method groups four points with very close pixel coordinates emerging in the grayscale and R-G-B images as the most representative keypoints. Thereafter, color synthesis for the feature descriptors is addressed by these four group points from four color images. Compared with color-invariant methods [57,58], the proposed method generates SC keypoints that can preserve the true color information in the image without deteriorating the spectral information. Therefore, the SC descriptors of the most representative keypoints are expected to be more distinctive than those obtained via the grayscale image alone. The arrangement of the SC descriptors is then modified by the ISR pattern, the cell sensitivity, and the distribution of color information across the human retina. Based on this mechanism, image matching can be carried out more efficiently by eliminating unlikely matches as early as possible. Figure 12 shows a linear fitting curve between the TC and the FPs obtained by the eight experimental examples (16 datasets analyzed with the EABRISK and SC-EABRISK operations) to estimate the processing time required when supplying different numbers of FPs. Although the estimation may be biased due to differences in the computer environments, the curve is approximated as TC = 0.000136 × FPs − 2.569 in this study, allowing prediction of the processing time needed when applying the proposed method to different imagery data.

There are two significant limitations to the proposed method. First, the number of SC keypoints may be very low in some specific cases, e.g., Figure 7b,c, caused by the discrete keypoints found in the four color images. It should be noted that too few SC keypoints may lead to both unsuccessful image matching due to a lack of feature correspondences and failure of the geometric mapping using affine transformation for achieving more feature correspondences. Therefore, further examination of the number of SC keypoints and their distribution across the image is recommended to ensure successful outcomes. The second limitation is related to the radiometric variation within the two images; for instance, Figure 7b and Figure 11b show that the result derived from SC-EABRISK weakens when the radiometric condition changes. In terms of this issue, Tsai and Lin [4] illustrated that the use of grayscale images is not influenced by the limitation of radiometric condition changes, and Ye et al. [82] also used such images to build structure features for multimodal image matching by the use of grayscale images. Based on the previous achievements, this paper suggests using the LATCH, ABRISK, EABRISK, feature structures, or BEBLID algorithm when the two images have drastic radiometric differences. Therefore, the developed SC-EABRISK with the ATBB method may be ineffective in coping with images of low temporal resolution (e.g., spanning month and year) due to unpredictable changes in the illumination conditions of the same region.

## 5. Conclusions

This paper proposes an integrated approach for improving the efficiency and performance of image matching based on BRISKs. In addition to using grayscale images, the proposed method adds color information extracted from R-G-B images to enhance the distinctiveness and robustness of the feature descriptors and improve the precision of the image matching. To suitably utilize the color information, the proposed method selects the keypoints that emerge in the four color spaces simultaneously and uses them as the most representative keypoints. For each of these representative keypoints, the 64-byte feature descriptors are rearranged following the mechanism underlying the human retina in terms of cell distribution and color recognition, and each keypoint in its corresponding color space contributes a portion of descriptors to form the SC feature descriptors. Every group containing four keypoints derived from the four color images is synthesized as an individual keypoint; thereafter, the EABRISK algorithm, which imitates visual accommodation, is applied to the SC feature descriptors for image matching, and thus, the SC-EABRISK algorithm aims to match the most representative keypoints and their more robust SC feature descriptors. The subsequent ATBB procedure further utilizes the results derived from the SC-EABRISK phase to extensively geometrically map the unused keypoints to find their likely correspondences. Both forward and backward geometric mapping thus involve all keypoints in the master and target images and the ATBB procedure allows the acquisition of a greater number of NCMs simply and effectively without additional TC.

The experimental results using benchmark imagery datasets, CRPs, and aerial and satellite images ensure the generalizability and practicability of the developed SC-EABRISK with ATBB method because the images were captured by different platforms and cameras. In terms of performance evaluation, this paper employed four indicators, the NCMs, MP, recall, and effectiveness, to assess the proposed method. Since the most representative keypoints are selected, the SC-EABRISK algorithm has a reduced number of NCMs and recall values, but the increased MPs and effectiveness values imply that image matching can be performed more precisely and efficiently. Following ATBB processing, the four indicators are significantly improved, indicating that most of the detected keypoints and their correspondences are found successfully. Therefore, all experimental outcomes indicate that the proposed method balances the NCMs and the TC, a profound issue when addressing image matching by previously proposed FBMs. Although the proposed method still presents some limitations, it is expected to improve the capacities of FBMs, leading to better spatial products and applications, such as image registration, SfM, and 3D reconstruction. For future works, the proposed SC-EABRISK algorithm may be extended to multispectral and hyperspectral satellite image matching by involving additional bands to make the feature descriptors more robust and distinctive. In addition, the image matching results may serve as training data for DL-based approaches to match additional images in the future.

## Figures and Tables

**Table 1:** Number of keypoints detected and feature pairs (FPs) of each benchmark image pair in the four color spaces.

Color Space	Grayscale		Red		Green		Blue		SC Keypoints	
Image pair	Dataset 1									
	master	target	master	target	master	target	master	target	master	target
Keypoints	1651	1568	1654	1576	1653	1577	1635	1564	1396	1407
FPs	2588768		-						1964172	
Image pair	Dataset 2									
	master	target	master	target	master	target	master	target	master	target
Keypoints	625	427	613	413	651	439	741	547	241	196
FPs	266875		-						47236	
Image pair	Dataset 3									
	master	target	master	target	master	target	master	target	master	target
Keypoints	1062	878	1034	804	1120	958	1013	913	363	311
FPs	932436		-						112893	
Image pair	Dataset 4									
	master	target	master	target	master	target	master	target	master	target
Keypoints	1644	753	1426	597	1819	926	2142	935	731	290
FPs	1237932		-						211990	

**Table 2:** Comparisons of the TC (seconds) by using the benchmark imagery datasets with different algorithms.

Algorithm	EABRISK	SC-EABRISK	SC-EABRISK with ATBB
Dataset 1	33.709	24.651	25.686
Dataset 2	4.180	0.637	1.398
Dataset 3	8.417	1.007	1.916
Dataset 4	10.686	1.773	2.362

**Table 3:** Number of keypoints detected and feature pairs (FPs) of each benchmark image pair in the four color spaces for CRPs.

Color Space	Grayscale		Red		Green		Blue		SC Keypoints	
Image pair	Drone image									
	master	target	master	target	master	target	master	target	master	target
Keypoints	2688	2270	2692	2509	2713	2327	2429	1997	1253	992
FPs	6101760		-						1242976	
Image pair	Ground-based CRPs									
	master	target	master	target	master	target	master	target	master	target
Keypoints	1712	2399	1999	2695	1621	2332	2200	2857	633	851
FPs	4107088		-						538683	

**Table 4:** Comparisons of the TC (seconds) by using CRPs with different image matching algorithms.

Algorithm	EABRISK	SC-EABRISK	SC-EABRISK with ATBB
Drone image pair	94.177	14.561	16.595
Ground-based CRP pair	44.54	4.677	6.834

**Table 5:** Number of keypoints detected and feature pairs (FPs) of each benchmark image pair in the four color spaces using aerial and satellite images.

Color Space	Grayscale		Red		Green		Blue		SC Keypoints	
Image pair	Aerial image									
	master	target	master	target	master	target	master	target	master	target
Keypoints	3361	2105	3594	2279	4013	2489	1591	1100	609	383
FPs	7074905		-						233247	
Image pair	Satellite image									
	master	target	master	target	master	target	master	target	master	target
Keypoints	2821	1093	2754	1094	2910	1102	2756	1097	1745	698
FPs	3083353		-						1218010	

**Table 6:** Comparisons of the TC (seconds) by using aerial and satellite image pairs with different algorithms.

Algorithm	EABRISK	SC-EABRISK	SC-EABRISK with ATBB
Aerial image pair	89.743	2.38	4.982
Satellite image pair	31.78	11.647	12.903

## Data Availability

Test imagery data can be accessed from https://www.robots.ox.ac.uk/~vgg/data/affine/ and https://www1.maths.lth.se/matematiklth/personal/calle/, accessed on 8 June 2021.

## References

[1] Liu Z., An J., Jing Y. (2012). A Simple and Robust Feature Point Matching Algorithm Based on Restricted Spatial Order Constraints for Aerial Image Registration. IEEE Trans. Geosci. Remote Sens..

[2] Gong M., Zhao S., Jiao L., Tian D., Wang S. (2014). A Novel Coarse-to-Fine Scheme for Automatic Image Registration Based on SIFT and Mutual Information. IEEE Trans. Geosci. Remote Sens..

[3] Ma W., Wen Z., Wu Y., Jiao L., Gong M., Zheng Y., Liu L. (2017). Remote Sensing Image Registration with Modified SIFT and Enhanced Feature Matching. IEEE Trans. Geosci. Remote Sens..

[4] Tsai C., Lin Y. (2017). An accelerated image matching technique for UAV orthoimage registration. ISPRS J. Photogramm. Remote Sens..

[5] Qin R., Greun A. (2014). 3D change detection at street level using mobile laser scanning point clouds and terrestrial images. ISPRS J. Photogramm. Remote Sens..

[6] Lerma J., Navarro S., Cabrelles M., Seguí A., Hernández D. (2013). Automatic orientation and 3D modelling from markerless rock art imagery. ISPRS J. Photogramm. Remote Sens..

[7] Cheng M.L., Matsuoka M. (2021). Extracting three-dimensional (3D) spatial information from sequential oblique unmanned aerial system (UAS) imagery for digital surface modeling. Int. J. Remote Sens..

[8] Ekhtari N., Javad M., Zoej V., Sahebi M., Mohammadzadeh A. (2009). Automatic building extraction from LIDAR digital elevation models and WorldView imagery. J. Appl. Remote Sens..

[9] Hartly R., Zisserman A. (2004). Multiple View Geometry in Computer Vision.

[10] Moulon P., Monasse P., Marlet R. Adaptive Structure from Motion with a Contrario Model Estimation. Proceedings of the Asian Computer Vision Conference (ACCV 2012).

[11] Sedaghat A., Mokhtarzade M., Ebadi H. (2011). Uniform Robust Scale-Invariant Feature Matching for Optical Remote Sensing Images. IEEE Trans. Pattern Anal. Mach. Intell..

[12] Stefano L., Mattoccia S., Tombari F. (2005). ZNCC-based template matching using bounded partial correlation. Pattern Recognit. Lett..

[13] Gruen A. (1987). Adaptive least square correlation: A powerful image matching technique. S. Afr. J. Photogramm. Remote Sens. Cartogr..

[14] Chen H., Varshney P., Arora M. (2003). Mutual information based image registration for remote sensing data. Int. J. Remote Sens..

[15] Remondino H., El-Hakim S., Gruen A., Zhang L. (2008). Turning images into 3-D models. IEEE Signal Process. Mag..

[16] Mikolajczyk K., Tuytelaars T., Schmid C., Zisserman A., Matas J., Schaffalitzky F., Kadir T., Gool L. (2005). A comparison of affine region detectors. Int. J. Comput. Vis..

[17] Li J., Allinson M. (2008). A comprehensive review of current local features for computer vision. Neurocomputing.

[18] Lowe D. (2004). Distinctive Image Features from Scale-Invariant Keypoints. Int. J. Comput. Vis..

[19] Mikolajczyk K., Schmid C. (2004). Scale and Affine Invariant Interest Point Detectors. Int. J. Comput. Vis..

[20] Hong G., Zheng Y. (2008). Wavelet-based image registration technique for high-resolution remote sensing images. Comput. Geosci..

[21] Yi K., Trulls E., Lepetit V., Fua P. Lift: Learned invariant feature transform. Proceedings of the European Conference on Computer Vision (ECCV 2016).

[22] Savinov N., Seki A., Ladicky L., Sattler T., Pollefeys M. Quad-networks: Unsupervised learning to rank for interest point detection. Proceedings of the IEEE Conference on Computer Vision and Pattern Recognition (CVPR’17).

[23] DeTone D., Malisiewicz T., Rabinovich A. Superpoint: Self-supervised interest point detection and description. Proceedings of the IEEE Conference on Computer Vision and Pattern Recognition Workshops (CVPRW’18).

[24] Ono Y., Trulls E., Fua P., Yi K. LF-NET: Learning local features from images. Proceedings of the 32nd International Conference on Neural Information Processing Systems.

[25] Wang S., Quan D., Liang X., Ning M., Guo Y., Jiao L. (2018). A deep learning framework for remote sensing image registration. ISPRS J. Photogramm. Remote Sens..

[26] Laguna A., Riba E., Ponsa D., Mikolajczyk K. Key.net: Keypoint detection by handcrafted and learned CNN filters. Proceedings of the IEEE International Conference on Computer Vision.

[27] Heipke C., Rottensteiner F. (2020). Deep learning for geometric and semantic tasks in photogrammetry and remote sensing. Geo-Spat. Inf. Sci..

[28] Ma J., Jiang X., Fan A., Jiang J., Yan J. (2021). Image Matching from Handcrafted to Deep Features: A Survey. Int. J. Comput. Vis..

[29] Chen L., Rottensteiner F., Heipke C. (2021). Feature detection and description for image matching: From hand-crafted design to deep learning. Geo-Spat. Inf. Sci..

[30] Liu Z., Monasse P., Marlet R. Match Selection and Refinement for Highly Accurate Two-View Structure from Motion. Proceedings of the European Conference on Computer Vision (ECCV 2014).

[31] Ham B., Cho M., Schmid C., Ponce J. Proposal Flow. Proceedings of the IEEE Conference on Computer Vision and Pattern Recognition (CVPR’16).

[32] Long J., Zhang N., Darrell T. (2014). Do Convnets Learn Correspondence?. Adv. Neural Inf. Process. Syst..

[33] Ufer N., Ommer B. Deep Semantic Feature Matching. Proceedings of the IEEE Conference on Computer Vision and Pattern Recognition (CVPR’17).

[34] Luo Z., Zhou L., Bai X., Chen H., Zhang J., Yao Y., Li S., Fang T., Quan L. Aslfeat: Learning local features of accurate shape and localization. Proceedings of the IEEE Conference on Computer Vision and Pattern Recognition (CVPR’20).

[35] Kanazawa A., Jacobs D., Chandraker M. WarpNet: Weakly Supervised Matching for Single-View Reconstruction. Proceedings of the IEEE Conference on Computer Vision and Pattern Recognition (CVPR’16).

[36] Choy C., Gawk J., Savarese S., Chandraker M. Universal Correspondence Network. Proceedings of the 30th International Conference on Neural Information Processing Systems.

[37] Zhou T., Krähenbühl P., Aubry M., Huang Q., Efros A. Learning Dense Correspondence via 3D-Guided Cycle Consistency. Proceedings of the IEEE Conference on Computer Vision and Pattern Recognition (CVPR’16).

[38] Revaud J., Weinzaepfel P., Harchaoui Z., Schmid C. (2016). DeepMatching: Hierarchical Deformable Dense Matching. Int. J. Comput. Vis..

[39] Bojanić D., Bartol K., Pribanić T., Petković T., Donoso Y., Mas J. On the Comparison of Classic and Deep Keypoint Detector and Descriptor Methods. Proceedings of the 2019 11th International Symposium on Image and Signal Processing and Analysis (ISPA).

[40] Bhowmik A., Gumhold S., Rother C., Brachmann E. Reinforced feature points: Optimizing feature detection and description for a high-level task. Proceedings of the IEEE Conference on Computer Vision and Pattern Recognition (CVPR’20).

[41] Xu W., Zhong S., Zhang W., Wang J., Yan L. (2021). A New Orientation Estimation Method Based on Rotation Invariant Gradient for Feature Points. IEEE Geosci. Remote Sens. Lett..

[42] Fan B., Kong Q., Wang X., Wang Z., Xiang S., Pan C., Fua P. (2019). A Performance Evaluation of Local Features for Image-Based 3D Reconstruction. IEEE Trans. Image Process..

[43] Liu J., Zhang L., Wang Z., Wang R. (2020). Dense Stereo Matching Strategy for Oblique Images That Considers the Plane Directions in Urban Areas. IEEE Trans. Geosci. Remote Sens..

[44] Sedaghat A., Ebadi H. (2015). Remote Sensing Image Matching Based on Adaptive Binning SIFT Descriptor. IEEE Trans. Pattern Anal. Mach. Intell..

[45] Ke Y., Sukthankar R. PCA-SIFT: A more distinctive representation for local image descriptors. Proceedings of the 2004 IEEE Computer Society Conference on Computer Vision and Pattern Recognition (CVPR’04).

[46] Mikolajczyk K., Schmid C. (2005). A performance evaluation of local descriptors. IEEE Trans. Pattern Anal. Mach. Intell..

[47] Bay H., Ess A., Tuytelaars T., Gool L. Speeded-Up Robust Features (SURF). Proceedings of the European Conference on Computer Vision (ECCV 2008).

[48] Yu G., Morel J. (2011). ASIFT: An Algorithm for Fully Affine Invariant Comparison. Image Process. Line.

[49] Wenger P. (1960). A technique for counting ones in a binary computer. Commun. ACM.

[50] Calonder M., Lepetit V., Strecha C., Fua P. BRIEF: Binary robust independent elementary features. Proceedings of the European Conference on Computer Vision (ECCV 2010).

[51] Rublee E., Rabaud V., Konolige K., Bradski G. ORB: An efficient alternative to SIFT or SURF. Proceedings of the 2011 International Conference on Computer Vision (ICCV’11).

[52] Leutenegger S., Chli M., Siegwart R.Y. BRISK: Binary Robust invariant scalable keypoints. Proceedings of the 2011 International Conference on Computer Vision (ICCV’11).

[53] Kamel M., Hussein S., Salama G., Elhalwagy Y. Efficient Target Detection Technique Using Image Matching Via Hybrid Feature Descriptors. Proceedings of the 2020 12th International Conference on Electrical Engineering (ICEENG).

[54] Liu Y., Zhang H., Guo H., Xiong N. (2018). A FAST-BRISK Feature Detector with Depth Information. Sensors.

[55] Cheng M.L., Matsuoka M. (2020). An Enhanced Image Matching Strategy Using Binary-Stream Feature Descriptors. IEEE Geosci. Remote Sens. Lett..

[56] Shao Z., Li C., Li D., Altan O., Zhang L., Ding L. (2020). An Accurate Matching Method for Projecting Vector Data into Surveillance Video to Monitor and Protect Cultivated Land. ISPRS Int. J. Geo-Inf..

[57] Abdel-Hakim A., Farag A. CSIFT: A SIFT Descriptor with Color Invariant Characteristics. Proceedings of the 2006 IEEE Computer Society Conference on Computer Vision and Pattern Recognition (CVPR’06).

[58] Jing H., He S., Han Q., Niu X. (2013). CBRISK: Colored Binary Robust Invariant Scalable Keypoints. IEICE Trans. Inf. Syst..

[59] Alitappeh R., Saravi K., Mahmoudi F. A New Illumination Invariant Feature Based on SIFT Descriptor in Color Space. Proceedings of the International Symposium on Robotics and Intelligent Sensors 2012 (IRIS 2012).

[60] Chen Y., Chan C., Tsai W. Creak: Color-based retina keypoint descriptor. Proceedings of the 2016 International Conference on Image Processing, Computer Vision, and Pattern Recognition (IPCV’16).

[61] Alahi A., Ortiz R., Vandergheynst P. FREAK: Fast Retina Keypoint. Proceedings of the 2012 IEEE Conference on Computer Vision and Pattern Recognition (CVPR’12).

[62] Hogan M., Weddell J. (1971). Histology of the Human Eye. An Atlas and Textbook.

[63] Ott M. (2006). Visual accommodation in vertebrates: Mechanisms, physiological response and stimuliu. J. Comp. Physiol. A.

[64] Garway-Heath D.F., Caprioli J., Fitzke F.W., Hitchings R.A. (2000). Scaling the hill of vision: The physiological relationship between light sensitivity and ganglion cell numbers. Investig. Ophthalmol. Vis. Sci..

[65] Watson A. (2014). A formula for human retinal ganglion cell receptive field density as a function of visual field location. J. Vis..

[66] Hendrickson A., Penfold P.L., Provis J.M. (2005). Organization of the Adult Primate Fovea. Macular Degeneration.

[67] Hartley R. (1997). In defense of the eight-point algorithm. IEEE Trans. Pattern Anal. Mach. Intell..

[68] Nïster D. (2004). An efficient solution to the five-point relative pose problem. IEEE Trans. Pattern Anal. Mach. Intell..

[69] Fischler M., Bolle R. (1981). A paradigm for model fitting with applications to image analysis and automated cartography. Commun. ACM.

[70] Torr P., Zisserman A. (1997). Robust parameterization and computation of the trifocal tensor. Image Vis. Comput..

[71] Subbarao R., Meer P. Beyond RANSAC: User Independent Robust Regression. Proceedings of the 2006 Conference on Computer Vision and Pattern Recognition Workshop (CVPRW’06).

[72] Chum O., Matas J., Kittler J. (2003). Locally Optimized RANSAC. Pattern Recognition.

[73] Raguram R., Chum O., Pollefeys M., Matas J., Frahm J.M. (2013). USAC: A Universal Framework for Random Sample Consensus. IEEE Trans. Pattern Anal. Mach. Intell..

[74] Barath D., Matas J. Graph-Cut RANSAC. Proceedings of the 2018 IEEE Conference on Computer Vision and Pattern Recognition (CVPR’18).

[75] Barath D., Matas J., Noskova J. MAGSAC: Marginalizing sample consensus. Proceedings of the 2019 IEEE Conference on Computer Vision and Pattern Recognition (CVPR’19).

[76] Mohammed H., El-Sheimy N. (2018). A Descriptor-less Well-Distributed Feature Matching Method Using Geometrical Constraints and Template Matching. Remote Sens..

[77] Jin Y., Mishkin D., Mishchuk A. (2021). Image Matching Across Wide Baselines: From Paper to Practice. Int. J. Comput. Vis..

[78] Mikolajczyk K., Schmid C. An Affine Invariant Interest Point Detector. Proceedings of the European Conference on Computer Vision (ECCV 2002) Lecture Notes in Computer Science.

[79] Maier J., Humenberger M., Zendel O., Vincze M. Ground truth accuracy and performance of the matching pipeline. Proceedings of the IEEE Conference on Computer Vision and Pattern Recognition Workshops (CVPRW’17).

[80] Levi G., Hassner T. LATCH: Learned arrangements of three patch codes. Proceedings of the IEEE Winter Conference on Applications of Computer Vision (WACV).

[81] Suárez I., Sfeir G., Buenaposada J., Baumela L. (2020). BEBLID: Boosted efficient binary local image descriptor. Pattern Recognit. Lett..

[82] Ye Y., Bruzzone L., Shan J., Bovolo F., Zhu Q. (2019). Fast and Robust Matching for Multimodal Remote Sensing Image Registration. IEEE Trans. Geosci. Remote Sens..

